# Exploring the recombinant evolution and hosts of crucivirus based on novel oyster-associated viruses

**DOI:** 10.3389/fmicb.2025.1454079

**Published:** 2025-02-04

**Authors:** Hong-Sai Zhang, Chang Liu, Guang-Feng Liu, Yu-Yu Chen, Peng Zhu, Xin Xu, Bing-Xin Yin, Jing-Zhe Jiang

**Affiliations:** ^1^College of Fisheries and Life Science, Shanghai Ocean University, Shanghai, China; ^2^Key Laboratory of South China Sea Fishery Resources Exploitation and Utilization, Ministry of Agriculture and Rural Affairs, South China Sea Fisheries Research Institute, Chinese Academy of Fishery Sciences, Guangzhou, China; ^3^Livestock, Aquaculture and Technology Promotion and Service Center of Conghua District, Guangzhou, China

**Keywords:** crucivirus, CRESS DNA viruses, virus evolution, host, genome, oyster

## Abstract

“Crucivirus” represents a group of viruses with chimeric genomes, significant for viral evolution and recombination studies. Their capsid proteins share homology with the RNA virus *tombusvirus*, while their replicase-associated proteins are homologous to a class of single-stranded DNA viruses, namely CRESS DNA viruses. This study identifies seven novel crucivirus genomes from oysters cultivated along the coast of the South China Sea. Phylogenetic analysis reveals that five sequences form a distinct branch, which may indicate the presence of a new subclass within the crucivirus family. We analyzed crucivirus from multiple perspectives, including viral genomes, hallmark proteins, sequence similarity, and potential hosts. The results indicate that the crucivirus genomes and replicase-associated proteins (Rep) from oysters conform to the typical characteristics of crucivirus; Crucivirus Rep appears to have a direct parallel origin from multiple clades of CRESS DNA viruses, while only the S-domain of their capsid proteins shows some evolutionary relationship with *tombusvirus*. We found protein sequences in rotifers that are highly similar to the Cap three-dimensional structure of crucivirus, which may suggest host relevance. Overall, this study provides new insights into the classification, evolution, and host origins of crucivirus.

## 1 Introduction

With the development of metagenomics, abundant viruses have been found in various environments. Due to the mobility of water, there are complex and diverse viral populations in water bodies, which play an important role in participating in aquatic biogeochemistry (Chow and Suttle, 2015; Simmonds et al., 2017; Tran Patricia and Anantharaman, 2021). As the most concentrated place of water resources on Earth, the ocean is a huge reservoir of virus resources. In 2019, Gregory et al. (2019) created the Global Marine DNA Virus Database (GOV 2.0, mainly dsDNA viruses infecting bacteria), which included 195,728 virus groups detected in 145 seawater samples collected from all over the world. In 2022, Zayed et al. (2022) reported 5,504 novel Marine RNA viruses, increasing the number of known phyla of RNA viruses from 5 to 10, and also found taxa that had been missing in the evolution of RNA viruses. Many studies have focused on viruses in seawater, while viruses in marine animals have been somewhat overlooked (Jiang et al., 2023). The surface, body, and blood of marine animals are teeming with viruses (Scanes et al., 2021). A herpes virus found in carp, for instance, can cause skin ulcers, organ damage, and other diseases in fish. This virus has been widely spread worldwide and is seriously impacting the marine ecosystem (Rakus et al., 2013).

As the exploration of the viral world progresses, an increasing number of new viruses are coming into view. Among these newly discovered viruses is a particularly intriguing group: the cruciviruses. In 2012, scientists first identified crucivirus in extreme environments, a novel hybrid virus suspected to be a recombinant of DNA and RNA viruses (Diemer and Stedman, 2012). This virus, closely related to CRESS DNA viruses (Circular Rep-encoding single-stranded DNA viruses), possesses a circular genome, a Rep gene, and the conserved stem-loop structure (ori), which is characteristic of the initiation site for CRESS DNA virus rolling-circle replication (Higuera et al., 2020). Interestingly, the capsid protein of crucivirus shares significant similarities with that of the RNA virus family *Tombusviridae* (Higuera et al., 2020). In general, this chimeric virus seems to have arisen from recombining two ostensibly unrelated DNA and RNA viruses. Studies have shown that recombination events frequently occur between cruciviruses and in replication related protein sequences, making their evolutionary relationships and origins more complex (Higuera et al., 2020). In addition, although crucivirus has been found in various natural environments including soil, lakes, and deep-sea sediments (Higuera et al., 2020), the specific functions and impacts of crucivirus in ecosystems are still poorly understood.

CRESS stands for Circular Rep-Encoding Single-Stranded. The CRESS DNA viruses possess circular ssDNA genomes ranging from 1,000 to 25,000 nucleotides, featuring two relatively conserved virus hallmark genes (VHGs): the capsid protein (Cap) and replicase-associated protein (Rep) (Rosario et al., 2012; Bistolas et al., 2017). Rep plays a role in the rolling-circle replication of single-stranded genomes. As a diverse group of viruses, CRESS DNA viruses infect a wide range of eukaryotic hosts, including metazoans, protozoans, plants, algae, fungi, and archaea, with no identified groups capable of infecting bacteria (Rosario et al., 2012). Thanks to high-throughput sequencing and metagenomics advancements, more CRESS DNA viruses have been discovered in environmental samples such as water, soil, air, and within organisms (Simmonds et al., 2017). Currently, the International Committee on Taxonomy of Viruses (ICTV) recognizes CRESS DNA viruses as comprising 12 families: *Bacilladnaviridae, Circoviridae, Geminiviridae, Genomoviridae, Metaxyviridae, Naryaviridae, Nanoviridae, Nenyaviridae, Redondoviridae, Smacoviridae, Amesuviridae, and Vilyaviridae* (Koonin et al., 2024).

Oysters are the world's largest cultivated shellfish and one of the important marine biological resources available to humans. Besides providing high-quality protein, oysters are also vital members of coastal ecosystems. Due to their widespread distribution and significant human impact, they have garnered extensive attention and serve as a “model species” in shellfish biology (Powell et al., 2018; Olalemi et al., 2016). Notably, as filter feeders, oysters subsist by filtering microorganisms from seawater, with a single oyster capable of filtering up to 5 liters per hour (Zhu et al., 2022). Many marine microorganisms, including viruses, accumulate within oysters, making them a natural repository for marine microbes and viruses. The Dataset of Oyster Virome (DOV) was reported by Jiang et al. (2023). DOV comprises 728,784 non-redundant viral operational taxonomic units (vOTUs) and 3,473 high-quality viral genomes, providing a comprehensive description of the oyster virome structure for the first time. A large number of unclassified CRESS-DNA viruses have been found in DOVs, which have been confirmed to be associated with aquatic animals and exhibit recombination in their genomes (Zhu et al., 2024). In this study, we delved into the DOV data, unexpectedly identifying seven strains of novel crucivirus. We conducted in-depth analyses from multiple aspects, including viral genomes, hallmark proteins, sequence similarity, and potential hosts, aiming to provide references for exploring the evolutionary origins of the crucivirus and potential hosts.

## 2 Result

### 2.1 Typical genomic features of crucivirus in oyster-related viruses

**Figure 1:**
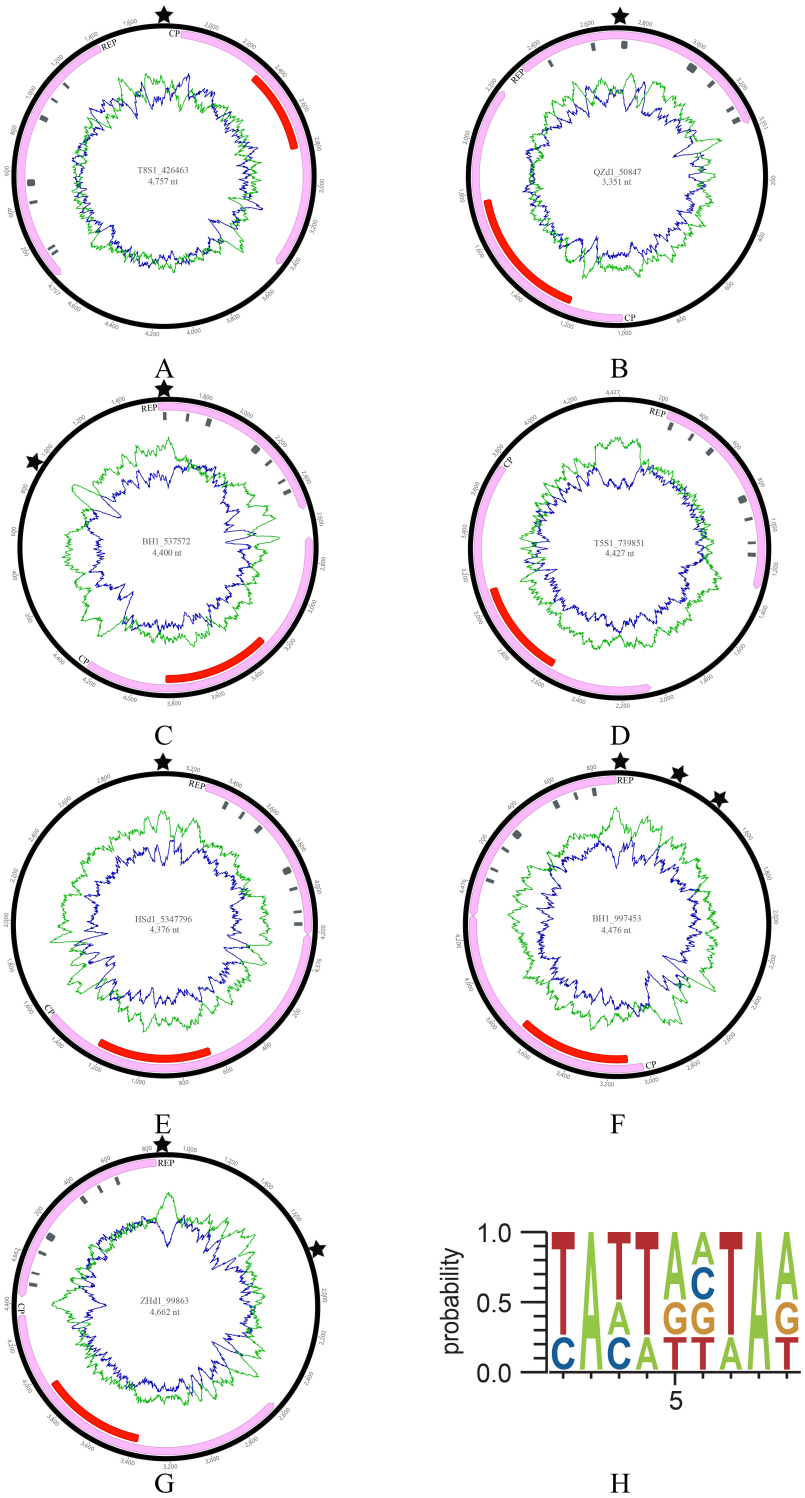
The genomic information of seven cruciviruses identified in oysters. **(A–G)** Represents the genome structure diagram. From the center of the circular diagram outward, the layers represent (1) the genome name (ID) and length (nt); (2) green and blue lines indicating the AT and GC content of the virus, respectively; (3) red rectangles representing the S-domain of Cap; gray rectangles along the direction of the Rep arrow representing the structural domains of Rep, including Motif I, Motif II, Motif III, Walker A, Walker B, Motif C, and Arg.f; (4) pink rectangles with arrows indicating the two ORFs (Cap and Rep) and their direction; (5) The black pentagram indicating the position of the replication initiation sites (ori). **(H)** Shows the sequence pattern of the ori.

In this study, seven cruciviruses were identified, with BH1_537572 and BH1_997453 originating from oyster (*Crassostrea hongkongensis*) samples cultured in Beihai, Guangxi Province; QZd1_50847 from Qinzhou, Guangxi; T8S1_426463 and T5S1_739851 from Taishan, Guangdong Province; ZHd1_99863 from Zhuhai, Guangdong; and HSd1_5347796 from oyster samples at the Huangsha seafood market in Guangzhou (Figure 1). These viral genomes exhibit typical crucivirus genomic features, including genome sizes ranging from 3,351 to 4,757 nt, GC contents between 35.4 and 43%, and two conserved open reading frames (ORFs), Cap and Rep (Figure 1). CRESS DNA viruses exhibit genome sizes ranging from 1.3 to 3 kb, whereas the genomes of oyster-related cruciviruses are larger than those of the CRESS DNA viruses currently known (Zhu et al., 2024). The Rep was annotated with seven conserved structural domains related to CRESS DNA viruses, including Motif I, Motif II, Motif III, Walker A, Walker B, Motif C, and Arg.f, depicted as gray rectangles in Figure 1. All seven genomes revealed a Cap protein homologous to *tombusvirus*, annotated with a conserved S-domain shown as red rectangles in Figure 1. However, the orientation of these virus-encoded ORFs varied, with QZd1_50847 and ZHd1_99863 being in the same direction (Figures 1B, G). In contrast, the remaining five were in opposite directions (Figures 1A, C–F). Additionally, except for T5S1_739851, which lacked the typical replication origin (ori) of CRESS DNA viruses genomes, the other viruses contained 1–3 oris (indicated by stars in Figure 1), consistent with the ori features of crucivirus reported by Higuera et al. (2020), all conforming to the NANTANTAN pattern (Figure 1H). Furthermore, In Higuera et al.'s study (Diemer and Stedman, 2012) one-third of the virus sequences could not predict Ori. Due to the fact that ori is the starting point of virus replication, and the genome of crucivirus exhibits high diversity through recombination events in replicase related proteins (Higuera et al., 2020), this may lead to the variability of ori.

### 2.2 Typical structural domains of CRESS DNA viruses rep in oyster crucivirus

**Figure 2:**
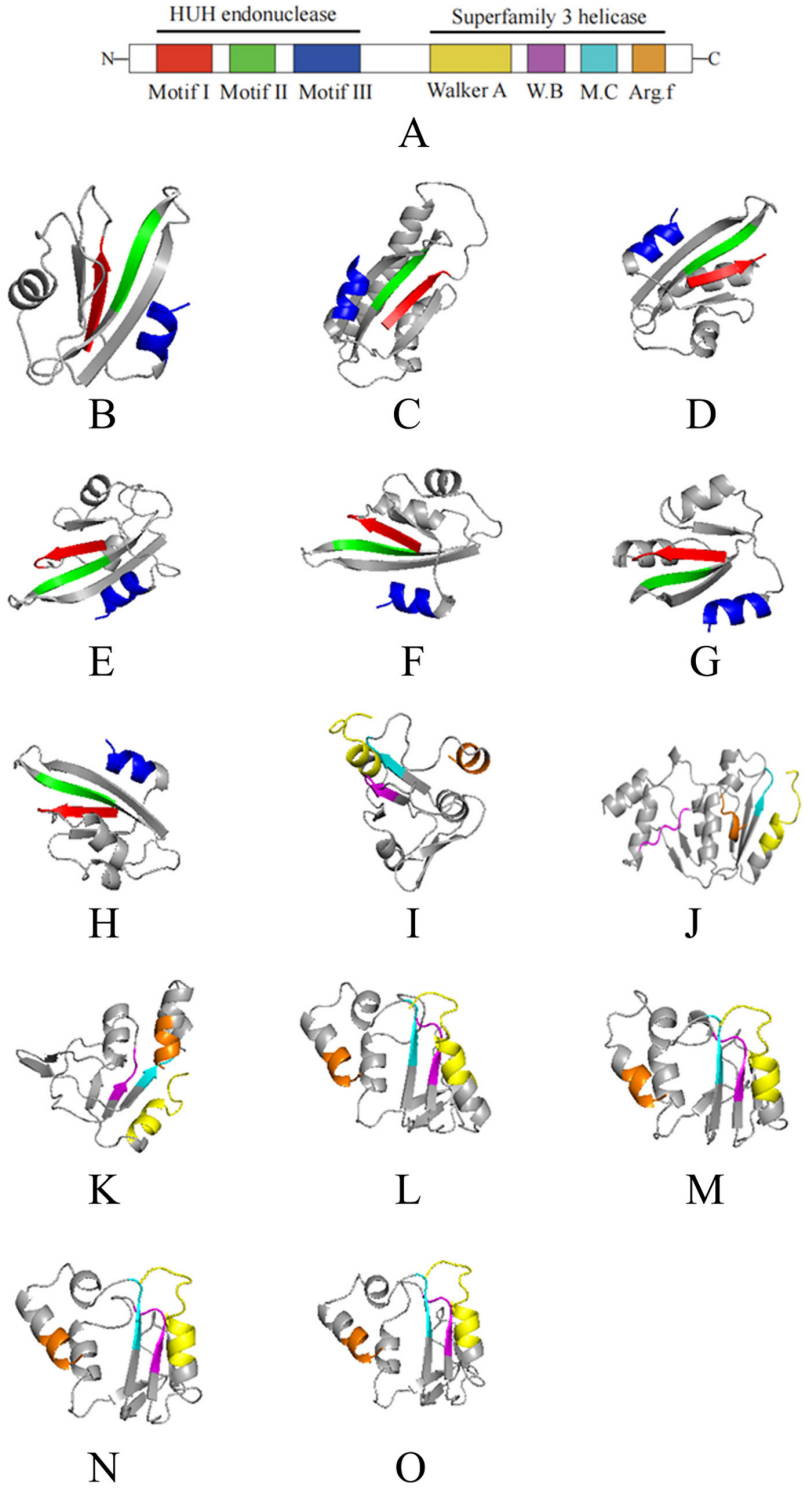
The 3D structural domains of the Rep protein of seven cruciviruses related to oysters. **(A)** is a schematic of the Rep structural domains, with different colors representing different domains. **(B–O)** correspond to the viruses T8S1_426463, QZd1_50847, BH1_537572, T5S1_739851, HSd1_5347796, BH1_997453, and ZHd1_99863, respectively, where **(B–H)** represent the HUH structural units of the seven Rep proteins, and **(I–O)** represent the S3H structural units of the Rep protein (in the same order as shown in Figure 1). The colors in **(B–O)** correspond to the domain colors in **(A)**, with red, green, blue, yellow, purple, cyan, and brown representing Motif I, Motif II, Motif III, Walker A, Walker B, Motif C, and Arg.f, respectively. The 3D structures were visualized using PyMol (1.8.6).

As shown in Figures 1, 2A, the Rep of the seven oyster-related cruciviruses was annotated with seven conserved structural domains of CRESS DNA viruses. Further, three-dimensional structure prediction using AlphaFold (Jumper et al., 2021) revealed high structural similarity among these Reps (Figures 2B–O), with Motif I, Motif II, Walker B, and Motif C primarily composed of β-sheets, and Motif III, Walker A, and Arg.f mainly composed of α-helices.

As a multifunctional protein, the Rep of CRESS DNA viruses possesses nuclease and helicase activities (Higuera et al., 2020), with Motif I, Motif II, and Motif III together forming the N-terminal HUH endonuclease domain (Figures 2B–H), which exhibits nuclease activity. The seven viruses discovered in this study all possess the typical features of the HUH domain: the conserved sequence of Motif I is “xxTxNN” (x represents any amino acid); the conserved sequence of Motif II is “xHxQG”, which plays a key role in maintaining nuclease activity during replication initiation (Koonin and Ilyina, 1992; Laufs et al., 1995b); the conserved sequence of Motif III is “YxxK”, involved in the cleavage of dsDNA during virus genome replication (Laufs et al., 1995a), with the lysine residue mediating binding and positioning in the catalytic process (Varsani and Krupovic, 2017; Vega-Rocha et al., 2007).

The helicase domain of CRESS DNA viruses Rep belongs to Superfamily 3 helicase (S3H) (Koonin, 1993), located at the C-terminal of Rep, composed of Walker A, Walker B, Motif C, and Arg.f (Figures 2I–O), which exhibits helicase activity (Gorbalenya et al., 1990; Choudhury et al., 2006). The seven viruses also possess the typical features of the S3H domain: the conserved sequence of the Walker A motif is “GxxxxGKT”, which recognizes ATP and binds to the conserved lysine residue (Rosario et al., 2012; Timchenko et al., 1999; Clérot and Bernardi, 2006); the conserved sequences of Walker B and Motif C are “uuDDu” and “uxxN” respectively (u represents a hydrophobic residue). The hydrophobic residues in the Walker B motif assist in ATP binding, crucial for ATP hydrolysis, while the conserved asparagine residue in the Motif C motif can interact with the γ-phosphate of ATP and the nucleophilic water molecule (Timchenko et al., 1999; George et al., 2014).

### 2.3 Multiple lineages of CRESS DNA viruses as direct ancestors of crucivirus rep

**Figure 3:**
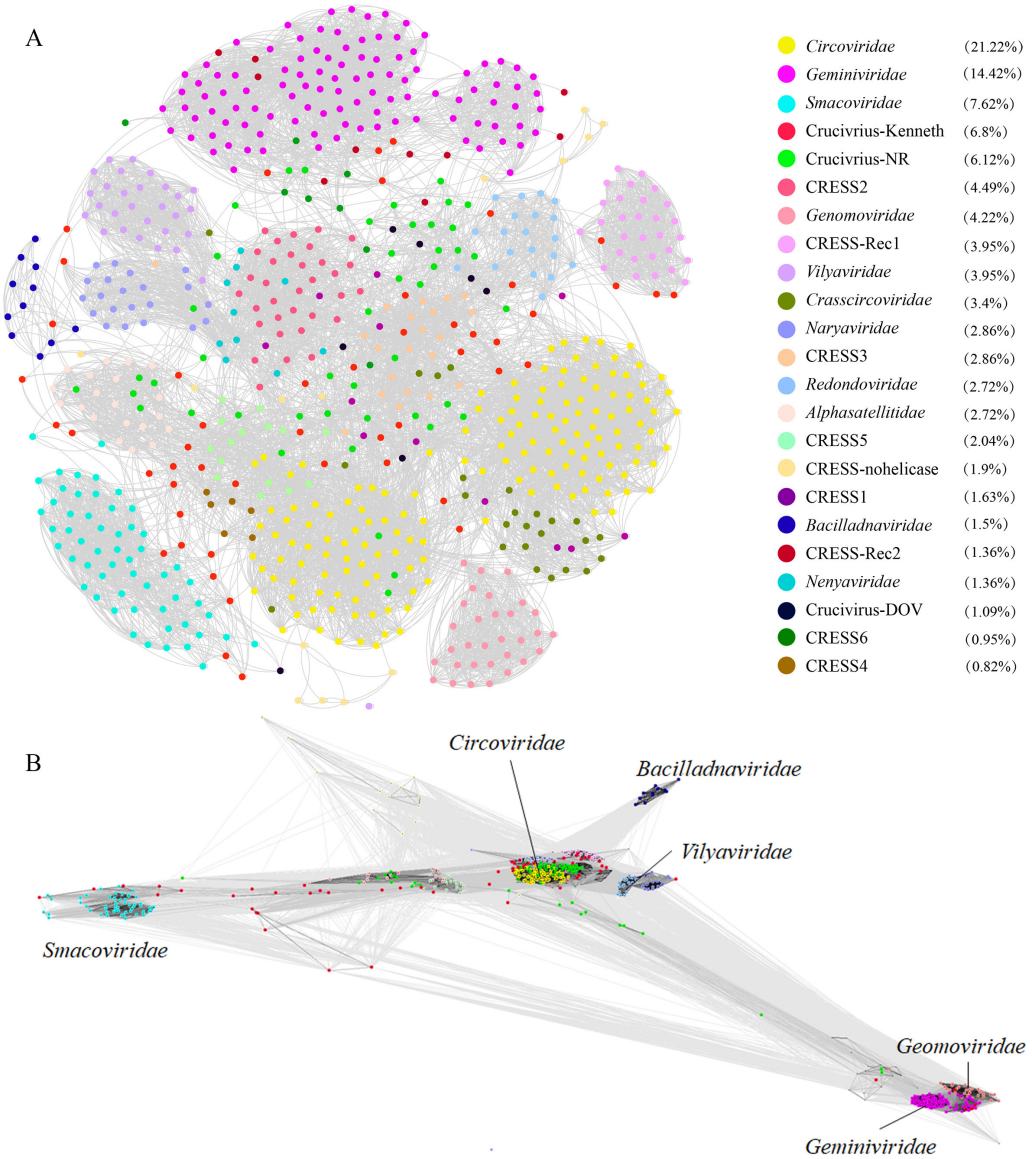
The sequence similarity clustering networks between the seven cruciviruses identified in oysters and related viruses with the Rep protein. **(A, B)** are clustering networks constructed using DIAMOND+gephi (Jiang et al., 2023; Zhu et al., 2022) and CLANS (Frickey and Lupas, 2004), based on the similarity of the virus Rep sequences (*n* = 721), with gray lines representing score values between two sequences, and colored dots representing different virus families. The numbers in the legend brackets indicate the proportion of viruses in each group, with Crucivirus-DOV representing the seven crucivirus sequences found in oysters, Crucivirus-NR representing sequences from the NCBI NR database similar to Crucivirus-DOV, Crucivirus-Kenneth representing crucivirus sequences reported by Kenneth Stedman (Higuera et al., 2020), and other sequences from ICTV and Krupovic (Kazlauskas et al., 2018).

To delve deeper into the evolutionary relationship between crucivirus and CRESS DNA virus Rep, we expanded our dataset to include NCBI NR-derived sequences similar to oyster crucivirus (Crucivirus-NR), 50 crucivirus sequences published by Higuera et al. (2020) (Crucivirus-Kenneth), and Rep sequences of CRESS DNA viruses classified by ICTV (https://ictv.global/) and published by Krupovic et al. (Kazlauskas et al., 2018) (*n* = 609). We employed two similarity clustering network methods, Diamond+Gephi (Jiang et al., 2023; Zhu et al., 2022) and CLANS, for the analysis. The results from Figures 3A, B collectively indicate that Crucivirus-DOV, Crucivirus-NR, and Crucivirus-Kenneth (Higuera et al., 2020) are intermixed with members of various CRESS DNA viruses families, with crucivirus Rep randomly distributed among the CRESS DNA virus lineages. The phylogenetic tree of Rep (Supplementary Figure S1) further supports the network diagram, showing a random distribution of crucivirus among the evolutionary branches of CRESS DNA viruses family members, with five and two oyster virus origins clustering in different evolutionary branches. This suggests that crucivirus shares the same set of Rep genes with CRESS DNA viruses, and crucivirus Rep does not originate from a specific CRESS DNA virus lineage but rather parallelly from multiple CRESS DNA viruses lineages (Supplementary Figure S5).

### 2.4 Structural differences of Cap between oyster crucivirus and tombusvirus

**Figure 4:**
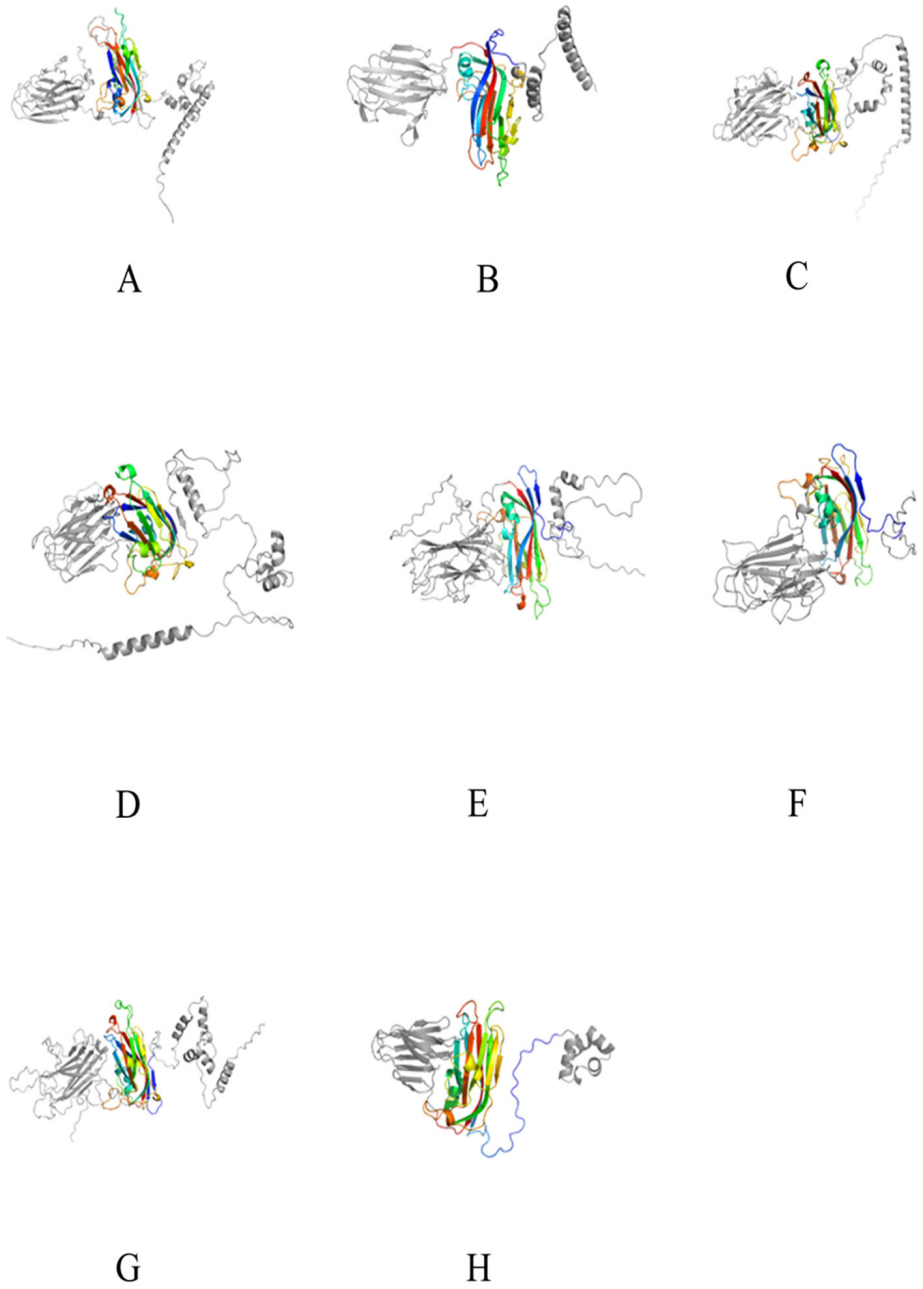
The 3D structure of the Cap protein of seven cruciviruses related to oysters. **(A–G)** represent the Cap protein structures of the viruses T8S1_426463, QZd1_50847, BH1_537572, T5S1_739851, HSd1_5347796, BH1_997453, and ZHd1_99863, respectively, and **(H)** represents the Cap protein structure of *Tombusviridae* (YP_009032648.1). The colored middle part represents the S-domain, and the gray parts on the left and right are the P-domain and R-domain, respectively. The 3D structures were visualized using PyMol (1.8.6).

The Cap of *Tombusviridae* typically includes three structural domains: the N-terminal R-domain facing the interior of the capsid protein, interacting with DNA; the middle S-domain facing the exterior of the capsid protein, containing a single-layer jelly-roll (SJR) structure, which is the basic structure of the capsid protein; and the C-terminal P-domain, a protrusion on the capsid protein, involved in interactions with the host (Gunawardene et al., 2017). Since the S-domain of the crucivirus Cap protein is more conserved in sequence than the P and R-domains (Higuera et al., 2020), only the S-domain was annotated by CDD (Wang et al., 2023). Further, according to the AlphaFold prediction results, the S-domain of the crucivirus Cap presents a single-layer jelly-roll structure consistent with the S-domain of *Tombusviridae* (Figure 4, colored structure) (Higuera et al., 2020). Further analysis of the RMSD values reveals the differences in three-dimensional structures. The RMSD values between the S-domains of crucivirus range from 0.182 to 0.698 (Figures 4A–G, colored structure), indicating a high degree of similarity or conservatism in their three-dimensional structures. In contrast, the RMSD values between crucivirus and *Tombusviridae* in the S-domain range from 0.688 to 2.848 (Figures 4A–H, colored structure), suggesting certain differences in the three-dimensional structure of the S-domain between these two viruses. Although the S-domain of crucivirus is predicted to adopt a single jelly roll (SJR) conformation similar to that of *Tombusviridae*, their specific spatial structures are not identical, which may reflect different evolutionary paths. Additionally, analysis of the three-dimensional structure of the P-domains shows an RMSD range from 0.77 to 21 (Figures 4A–H, left gray structure), indicating significant structural diversity and potentially different evolutionary histories among the P-domains. In summary, the Cap of crucivirus shows differences from *Tombusviridae* at both the sequence and three-dimensional structure level.

### 2.5 Crucivirus Cap does not directly originate from tombusvirus

**Figure 5:**
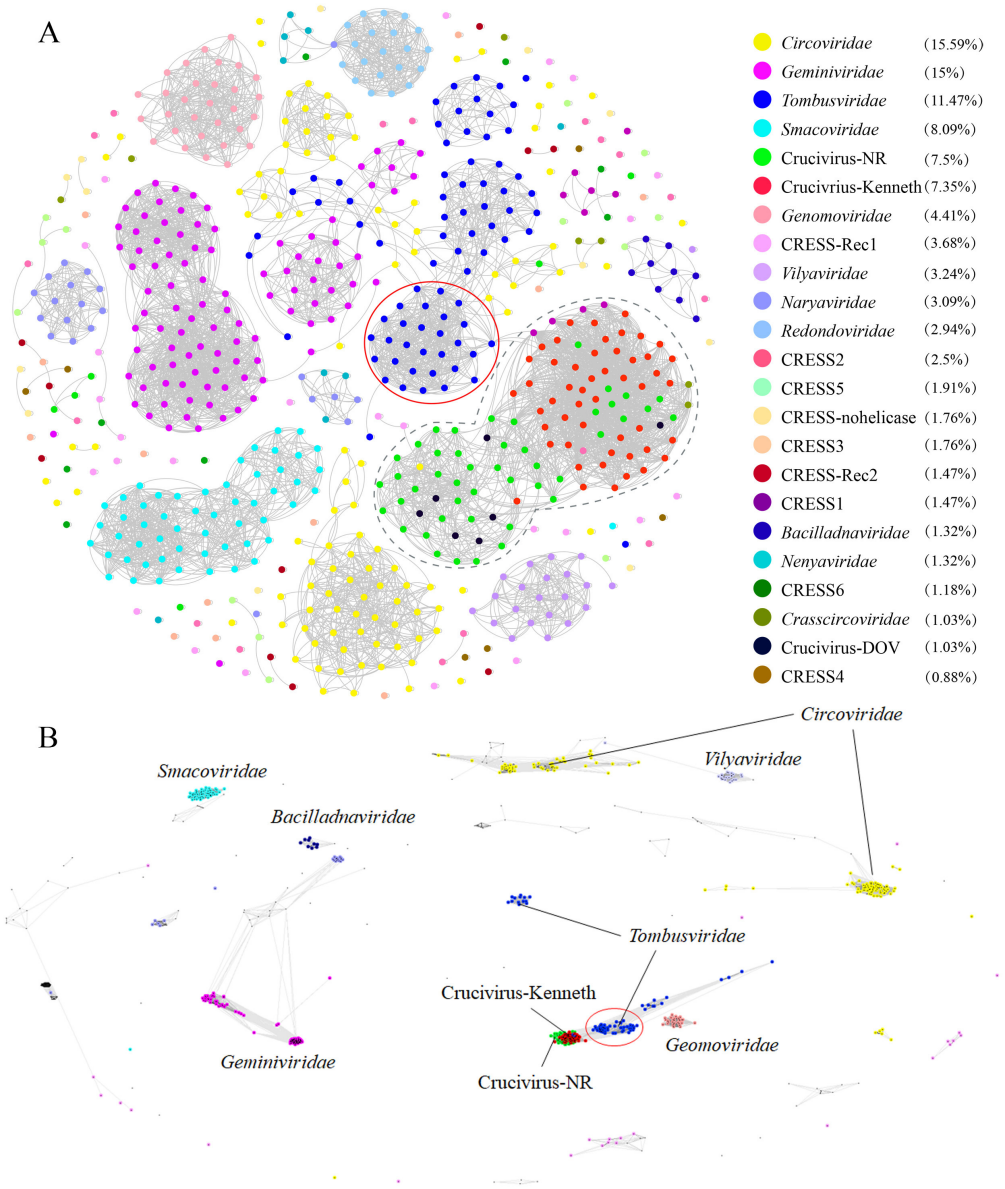
The sequence similarity clustering networks between the seven cruciviruses found in oysters and related viruses with the Cap protein. **(A, B)** are clustering networks constructed using DIAMOND+gephi (Jiang et al., 2023; Zhu et al., 2022) and CLANS (Frickey and Lupas, 2004). Both tools used the same Cap dataset (*n* = 734). Gray lines represent the score values between two sequences, and colored dots represent different virus families. The numbers in the legend brackets indicate the proportion of viruses in each group, with Crucivirus-DOV representing the seven crucivirus sequences found in oysters, Crucivirus-NR representing sequences from the NR database similar to Crucivirus-DOV, Crucivirus-Kenneth representing crucivirus sequences reported by Kenneth Stedman (Higuera et al., 2020) and other sequences from ICTV and Krupovic (Kazlauskas et al., 2018).

We also employed the two clustering methods from 2.3 to analyze the clustering relationship of the Cap proteins of the crucivirus-related virus family. As shown in Figure 5, Crucivirus-DOV (black dots), Crucivirus-NR data (green dots), and Crucivirus-Kenneth (red dots) all form a relatively tight cluster. This cluster is closely related to the *Procedovirinae* subfamily of *Tombusviridae* (red circle) but still at a certain distance. Notably, five Crucivirus-DOV and Crucivirus-NR sequences form a relatively independent cluster (Figure 5A), distinctly separated from Crucivirus-Kenneth (red dots). We constructed a phylogenetic tree of oyster-derived and NR-derived crucivirus (Supplementary Figure S2), revealing that crucivirus in oysters is divided into two major branches, with five oyster viruses located in the lower branch. This result is consistent with Supplementary Figure S1, indicating that these five oyster viruses represent a new subcategory of crucivirus.

### 2.6 Origin of the S-domain in crucivirus Cap

**Figure 6:**
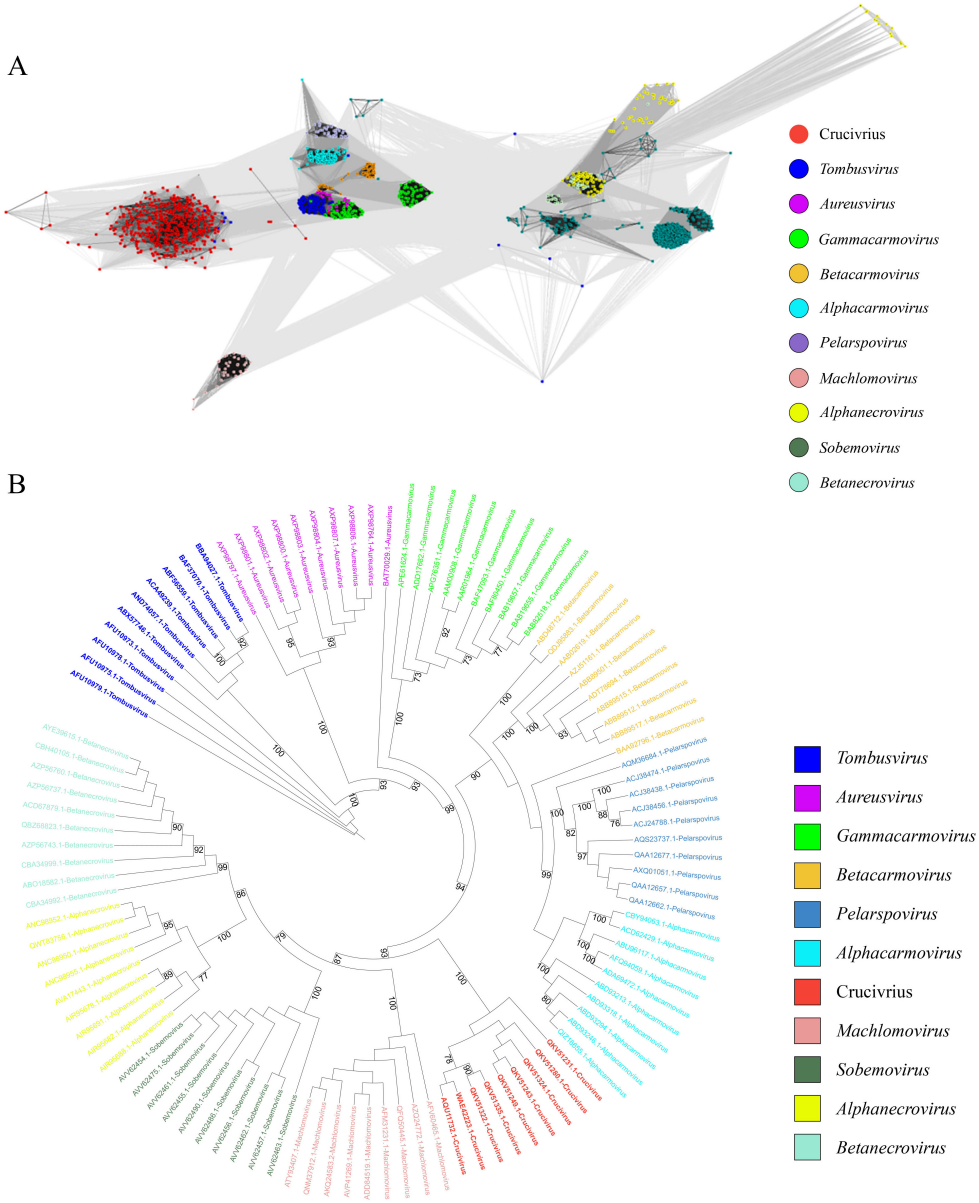
The evolutionary tree of the crucivirus capsid protein. **(A)** shows different colors representing different virus capsid protein families, with gray lines indicating score values between two sequences. **(B)** is an evolutionary tree constructed using the S-domain, with different colors representing different virus branches.

To further explore the evolutionary origin of the crucivirus capsid protein, we used the most conserved S-domain of crucivirus Cap to search the nr30 database with HMMER (Potter et al., 2018), obtaining all sequences with the S-domain, resulting in 1,067 virus sequences from nine genera within the family *Tombusviridae* and sobemovirus. We constructed a CLANS network diagram with the above data and crucivirus (*n* = 457) (Figure 6A). The results show that the capsid protein of crucivirus (red dots) represents a separate group, which is relatively close to *tombusvirus* (blue dots, belonging to *Procedovirinae*) (Figure 6A), consistent with the results of Figure 5. Further, we selected ten capsid protein sequences of the S-domain from each virus genus to construct an evolutionary tree (Figure 6B). The S-domain of *tombusvirus* (blue branch) is located at the ancestral branch at the tree's base. In contrast, the crucivirus (red branch) is located in a separate branch in the middle-lower part of the tree. This branch is parallel to *machlomovirus, sobemovirus, betanecrovirus*, and alphanecrovirus, indicating that the time when crucivirus acquired the S-domain is close to the evolutionary time of these virus groups. Combining the distances between the clusters in Figure 6A and the evolutionary relationships of the branches in Figure 6B, it can be inferred that the S-domain of *tombusvirus*, as the center of S-domain evolution, first evolved into the closely related branches of *aureusvirus* and *gammacarmovirus*, then further derived the *batacarmovirus*-*alphacarmovirus*-*pelarspovirus* branch, and finally derived the crucivirus-*machlomovirus*-*sobemovirus*-*betanecrovirus*-*alphanecrovirus* branch.

### 2.7 Didymodactylos-related groups may be hosts of crucivirus

**Figure 7:**
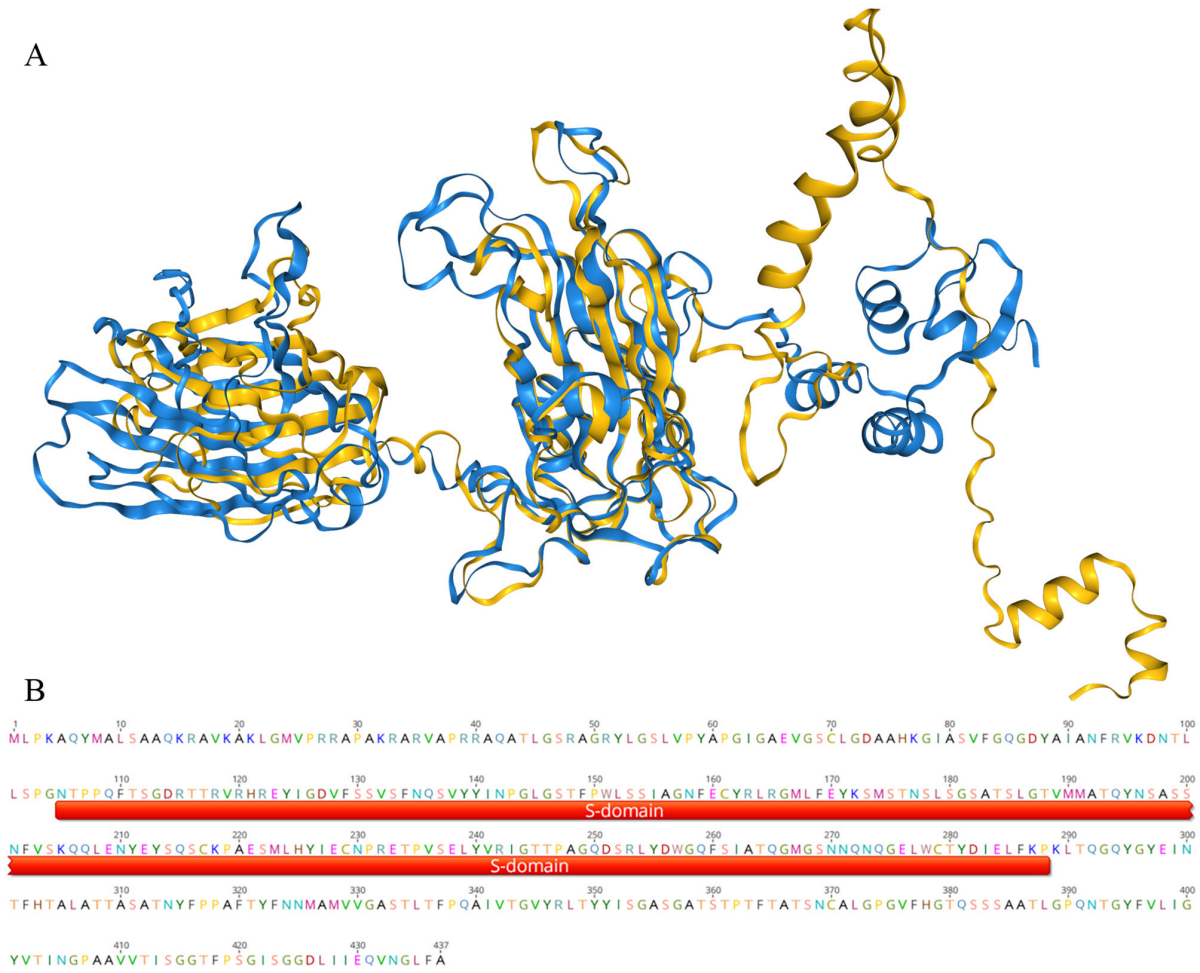
The three-dimensional structure comparison of the crucivirus Cap protein. In **(A)**, the blue portion represents the three-dimensional structure of the crucivirus capsid protein (T8S1-426463), and the yellow represents the three-dimensional structure of a protein sequence from *Didymodactylos carnosus* (CAF1628476.1). **(B)** shows a segment of the protein CAF1628476.1, with the red portion indicating the annotated S-domain within this sequence.

After infecting a host, there is a certain probability that a virus will integrate its genome or part of its sequence into the host genome (Carey et al., 2019). So, finding sequences related to the virus in the host genome can provide evidence for establishing the connection between the virus and the host. To find clues about the host of crucivirus, we first searched for similar sequences in non-viral organisms using BLAST with the Rep and Cap nucleotide and protein sequences, but no significant hits were found. We then attempted a more conservative three-dimensional structure comparison method, using Foldseek Search (van Kempen et al., 2024) to find structural similarity in the structural database. As depicted in Figure 7, a protein from *Didymodactylos carnosus* (CAF1628476.1) exhibits a highly similar structure to the S-domain of crucivirus (RMSD = 0.567). Furthermore, although the RMSD between the P-domains of the two structures is 13.8, indicating a significant difference, direct observation of Figure 7A reveals that there appears to be some degree of similarity in the P-domains. *D. carnosus* is a rotifer belonging to the family *Philodinidae* and genus *Didymodactylos*, which is widely found in both freshwater and seawater. These findings suggest that rotifers and other eukaryotic microorganisms are clues for searching for crucivirus hosts.

## 3 Discussion

Cruciviruses represent a highly valuable group of viruses characterized by genomes comprising sequences derived from DNA and RNA viruses. Since the first crucivirus sequence was discovered in 2012, over 500 crucivirus genomes have been reported. The known crucivirus genomes vary in size, ranging from 2.7 to 5.7 kb, and typically contain additional open reading frames beyond the Rep and Cap proteins. This class of viruses is commonly found in aquatic environments such as lakes, rivers, and even deep-sea sediments (Higuera et al., 2020). In an unexpected finding from DOV data sourced from oyster tissues, this study identified seven new strains of crucivirus, marking the first discovery of crucivirus within aquatic animals, thereby expanding the known habitats of crucivirus. Notably, both the Rep (Supplementary Figure S1) and Cap (Supplementary Figure S2) phylogenetic results show that five virus sequences from oysters and related NR virus sequences form distinct, independent branches, emphasizing that oyster-related crucivirus may contain unique taxa. These results are similar to studies on other oyster-related CRESS DNA viruses (Zhu et al., 2024; Jiang et al., 2023). In addition, in the study of oyster related CRESS DNA viruses, oysters contain a large number of novel CRESS DNA viruses, which are significantly different from viruses in marine environments or other habitats. It has been found that the replication related proteins and capsid proteins of these CRESS DNA viruses exhibit different evolutionary rates and often undergo recombination. In Figure 3, it can be observed that the Rep protein of Crucivirus exhibits high diversity. Studies suggest that genetic recombination is a primary factor contributing to the diversity observed in crucivirus Rep (Higuera et al., 2020). Additionally, frequent recombination is identified as an important determinant of variation. This suggests that intragenic recombination plays a significant role in the adaptive evolution of crucivirus. The distinct habitats provided by marine animals for marine viruses, as compared to the broader marine environment, are also highlighted. Investigating and analyzing viruses associated with marine animals will contribute to a more comprehensive understanding of viral taxonomy and evolution.

With the advancement of metagenomic sequencing technology, numerous novel viral genome sequences are being discovered (Simmonds et al., 2017). Although the ICTV encourages genome-based viral classification (Simmonds et al., 2017), the current classification standards based on a few VHGs do not apply to all viruses. For instance, the classification of cruciviruses is challenging, with ongoing debates over whether to prioritize proteins involved in viral replication over structural proteins for classification purposes (Krupovič and Bamford Dennis, 2010). Cruciviruses have circular genomes, and their replication-associated proteins fully conform to the characteristics of CRESS DNA viruses. If classified according to the Rep-based method for CRESS DNA viruses (Rosario et al., 2012), the crucivirus would belong to CRESS DNA viruses. However, this overlooks the evolutionary information of another important structural gene, Cap, in crucivirus. This study employed two clustering methods, DIAMOND+gephi (Jiang et al., 2023; Zhu et al., 2022) and CLANS (Frickey and Lupas, 2004), for comparative analysis of Rep and Cap, demonstrating that crucivirus and CRESS DNA viruses share the same set of Rep, making it impossible to distinguish between the two viruses based on Rep alone. The Cap network diagram reveals that the Cap of cruciviruses lacks similarity to the Cap of CRESS DNA viruses and also shows significant differences from the Cap of *tombusvirus* (Figure 5), suggesting that crucivirus did not simply integrate the Cap gene from *tombusvirus* but may originate from an as-yet-undiscovered RNA virus family with a conserved S-domain. This RNA virus likely coexists extensively with various CRESS DNA viruses lineages within marine animals like oysters or other aquatic environments, independently undergoing multiple gene recombination events at opportune times and evolving into multiple crucivirus lineages in parallel. Given the distinct evolutionary features of crucivirus from the CRESS DNA viruses group and its pivotal evolutionary link between CRESS DNA viruses and S-domain-related RNA viruses, we hope that our research can provide a reference for the classification of crucivirus by the ICTV.

This study provides two important insights: first, viral classification and evolution should not solely focus on evolutionarily conserved replication-associated genes such as Rep or RdRp. Frequent and diverse structural genes, other important functional genes, and even unique recombination processes of some viruses should also be considered in viral classification, otherwise, a large number of “special” viruses like crucivirus would remain undescribed. Second, the complex structural pattern of crucivirus Cap indicates that the rapid mutation rate and adaptability of viruses stem not only from genomic sequence variation and rapid intergenic recombination but also from intragenic recombination, particularly within different structural domains of the capsid protein, which plays a non-negligible role in enhancing viral adaptability and host jumping mutations. This aspect warrants focused attention in the in-depth study of crucivirus evolution.

Artificial infection experiments can directly determine the host of a virus, but this method is impractical for the vast number of viruses identified through metagenomics. With the exponential expansion of viral sequences, there is an urgent need to establish high-throughput, bioinformatics-based methods for predicting viral hosts. The advent of AlphaFold (Jumper et al., 2021) has undoubtedly revolutionized biological research. The AlphaFold tool has determined the structures of approximately 200 million proteins from almost all known organisms on Earth. Unlike time-consuming and expensive experimental methods such as X-ray crystallography and cryo-electron microscopy, AlphaFold simplifies structural analysis of a massive number of unknown proteins, akin to using a search engine. In this study, we used a method based on three-dimensional protein structure comparison to find distant similarities, suggesting that *Didymodactylos carnosus* may be the host of cruciviruses. *Didymodactylos carnosus* is a rotifer widely present in both freshwater and seawater, consistent with the observation that cruciviruses are often found in aquatic environments. Other research (Higuera et al., 2020) has found that the codon usage characteristics of crucivirus are similar to those of ciliates, and crucivirus has been discovered in locations where isopod ectoparasites are present. Since ciliates and isopod ectoparasites belong to the SAR supergroup, it is speculated that the host of crucivirus comes from the SAR supergroup. Rotifers and ciliates are similar in body length, with the former being about 440 micrometers and the latter ranging from tens to hundreds of micrometers. Both feed on microorganisms such as algae and share similar living environments. However, whether the host of crucivirus is rotifers or SAR-class eukaryotic microorganisms requires further support from bioinformatics and experimental evidence.

## 4 Conclusion

This study reports seven crucivirus viral genomes from aquatic animals, providing an in-depth analysis of these cruciviruses in five aspects: genomic structure, the three-dimensional structure of hallmark proteins, similarity clustering networks, the evolutionary origin of the S-domain in capsid proteins, and potential hosts. The results significantly expand the known sources and diversity of crucivirus, propose possible evolutionary and recombination pathways for crucivirus, offer new insights into the classification of recombinant viruses like crucivirus, and make preliminary explorations into their hosts and ecological significance, enriching our understanding of crucivirus.

## 5 Materials and methods

### 5.1 Sequence assembly and virus discovery

We constructed 54 oyster virome libraries from various sources, including 9 time points, seven locations (Qinzhou, Yangjiang, Zhuhai, Huidong Tanwei area, Lianjiang, Shenzhen in Guangxi), and two tissue types (Jiang et al., 2023). Through virome sequencing of oysters cultured in many areas along the southern coast of China, we obtained ~2.5 billion reads. We used fastp (version 0.20.0) (Chen et al., 2018) for quality control by removing low-quality sequences and adapters, and assembled the reads into contigs using MEGAHIT (version 1.2.9) (Li et al., 2015, 2016). Sequences were aligned and annotated using DIAMOND (version 0.9.24.125) (Buchfink et al., 2015) with the non-redundant protein (nr) database from the National Center for Biotechnology Information (NCBI) as a reference. Please refer to the research of Jiang et al. (2023) for specific parameters. Finally, seven viral genome sequences were identified as cruciviruses and selected for further in-depth analysis.

### 5.2 Open reading frame prediction and annotation

The seven viral genomes' open reading frames (ORFs) were predicted and annotated using Prodigal (Hyatt et al., 2010). ORF sequences were aligned with the nr database using NCBI BLASTP (Altschul et al., 1997) with an e-value cutoff set to 10^−5^. The highest consistency protein sequences were reverse-aligned with the viral genome sequences using NCBI tBLASTN (Altschul et al., 1997) to verify the completeness of the ORF predictions.

### 5.3 Similarity clustering analysis

Based on the total score of BLASTP results, we selected the top 10 Rep sequences and top 10 capsid protein sequences and aligned them using DIAMOND (Buchfink et al., 2015). We then constructed clustering networks based on scores using Gephi (Bastian et al., 2009) (version 0.9.2). Only scores higher than 150 are shown. CLANS (Frickey and Lupas, 2004) implements the Fruchterman-Reingold force-directed layout algorithm, which treats protein sequences as point masses in a virtual multidimensional space, where protein sequences attract or repel each other based on the strength of their pairwise similarities. Scoring Matrix chooses BLOSUM62, Extract BLAST HSP's up to E-values of 1e^−4^. We performed CLANS clustering using the Bioinformatics Toolkit (Alva et al., 2016) online tool, a free one-stop web service for protein bioinformatics analysis provided by the MPI Bioinformatics Toolkit (https://toolkit.tuebingen.mpg.de). It currently offers 34 interconnected external and internal tools, covering functions such as sequence similarity search, alignment construction, sequence feature detection, structure prediction, and sequence classification (Gunawardene et al., 2017; van Kempen et al., 2024).

### 5.4 Viral genome structure analysis and visualization

(1) Annotation of the S-domain was done using CDD (Wang et al., 2023) (https://www.ncbi.nlm.nih.gov/Structure/cdd), and motifs in Rep were annotated using PSI-Coffee (http://tcoffee.crg.cat/). The viral genomes were annotated and visualized using Geneious Prime 2023.0.4. (2) The three-dimensional structures of the viruses were predicted using AlphaFold (Jumper et al., 2021) then visualized and annotated using PyMol (1.8.6). In the predicted results, about 80% of the area is displayed in blue (pLDDT > 70) (3) Three-dimensional structure comparison was performed using Foldseek Search (van Kempen et al., 2024) (https://search.foldseek.com/search) (4) Sequence logos displaying ori base frequencies were created using the WebLogo server (http://weblogo.threeplusone.com/).

### 5.5 Homology search for S-domain

In the process of searching for viruses with the S-domain, we first used the S-domain of crucivirus to find homologous proteins in the nr30 database using HMMER (Potter et al., 2018), set the cutoff value to 1e-3, then expanded the homologous protein families in Pfam (Pfam: PF00729) (Mistry et al., 2021) and InterPro (Paysan-Lafosse et al., 2023). All virus sequences were then clustered using CLANS. HMMER (Potter et al., 2018) tool was also accessed through the Bioinformatics Toolkit (Alva et al., 2016).

### 5.6 Phylogenetic tree construction based on Rep and Cap sequences

Multiple sequence alignments were performed using MAFFT (Katoh and Standley, 2013), ambiguous regions were removed using trimAL (Capella-Gutiérrez et al., 2009), and maximum likelihood phylogenetic trees based on Rep and Cap protein sequences were constructed using IQ-TREE (version 2.1.4) (Minh et al., 2020). ModelFinder was set to MFP (forModelFinder Plus) and 10,000 ultrafast bootstrap replicates were used. Visualization was done using iTOL (version 6.5.2) (https://itol.embl.de) (Letunic and Bork, 2016).

## Data Availability

The datasets presented in this study can be found in online repositories. The names of the repository/repositories and accession number(s) can be found below: https://www.cncb.ac.cn/, C_AA071460-66.
