# Supplementary material for: Exploring the recombinant evolution and hosts of crucivirus based on novel oyster-associated viruses
Source: Front Microbiol. 2025 Feb 4;16:1454079. doi: 10.3389/fmicb.2025.1454079 (PMC11832652; doi:10.3389/fmicb.2025.1454079)
Supplement: Supplementary file 1 [file Data_Sheet_1.doc]

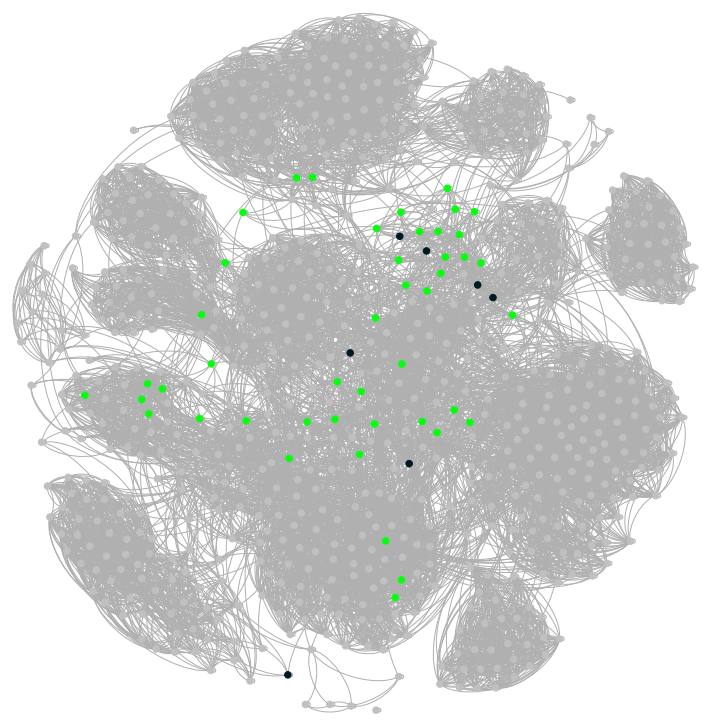


A


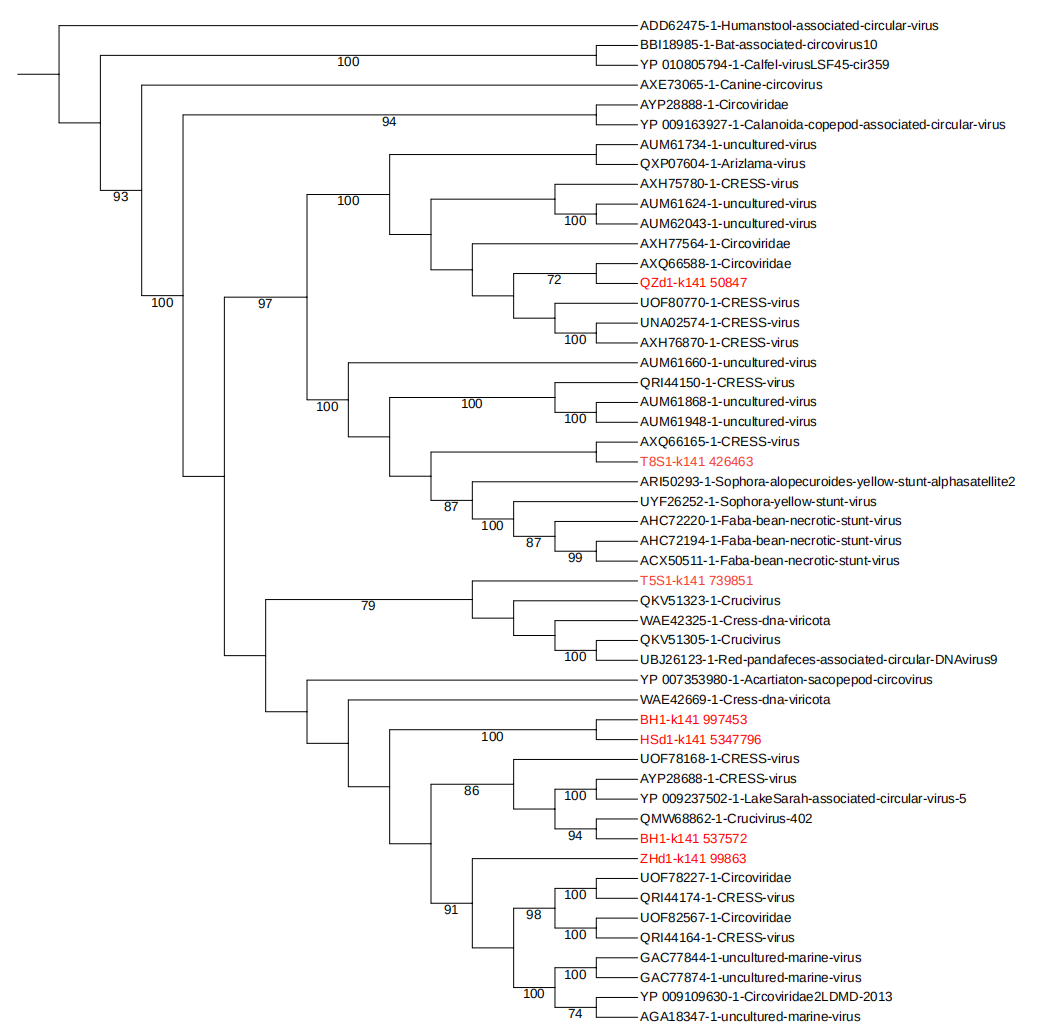


B

Figure S1 The sequence similarity clustering network for 7 Crucivirus and related viruses found in oysters, in relation to the Rep protein of CRESS DNA viruses, constructed using Gephi. Grey lines indicate a Score value between two sequences. The green and black colors in the figure represent the Crucivirus-NR dataset and the Crucivirus-DOV dataset, respectively. These were selected for the construction of the phylogenetic tree in Figure B. The sequence at the top end of the phylogenetic tree is the outgroup we selected, with the red sequences representing Crucivirus-DOV.
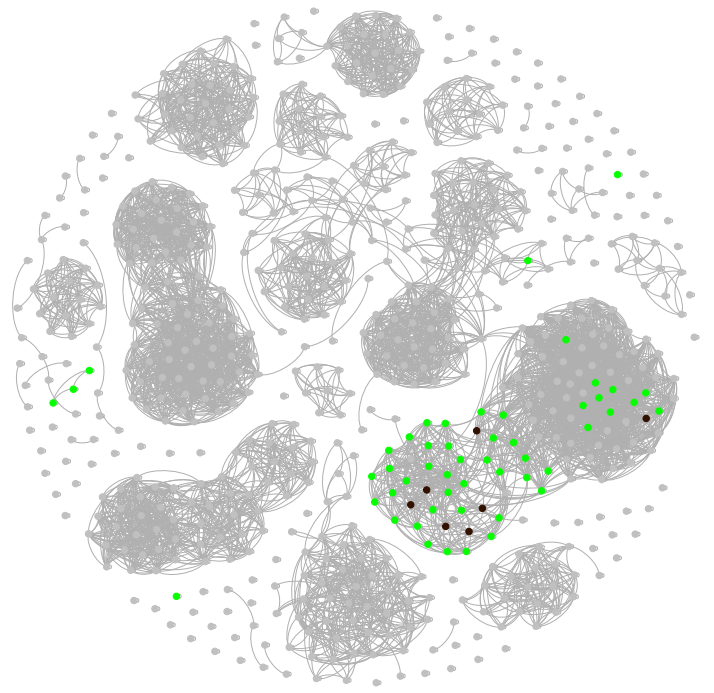


A


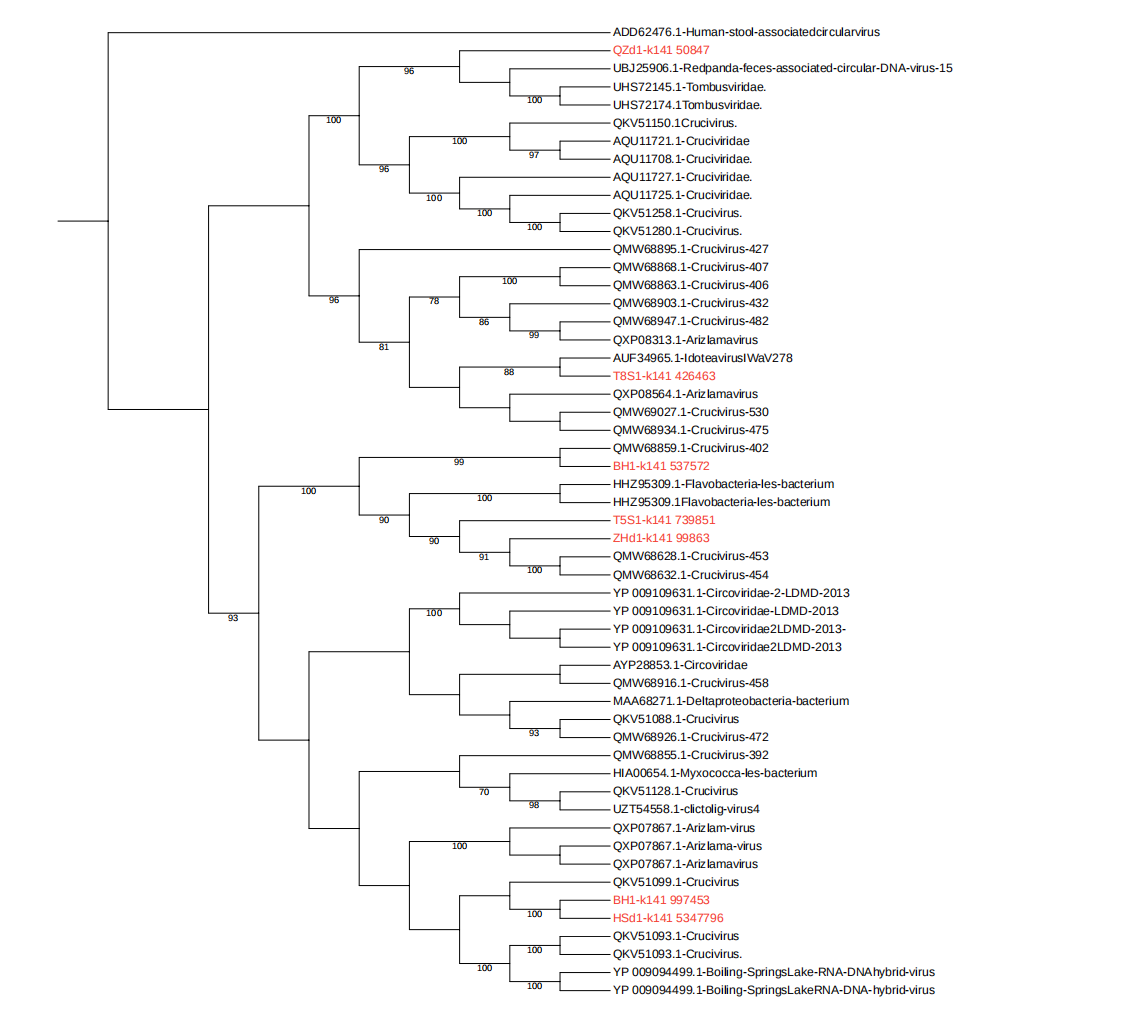


B

Figure S2 The sequence similarity clustering network for 7 Crucivirus and related viruses found in oysters, in relation to the Cap protein of CRESS DNA viruses, constructed using Gephi. Grey lines indicate a Score value between two sequences. The green and black colors in the figure represent the Crucivirus-NR dataset and the Crucivirus-DOV dataset, respectively. These were selected for the construction of the phylogenetic tree in Figure B. The sequence at the top end of the phylogenetic tree is the outgroup we selected, with the red sequences representing Crucivirus-DOV.


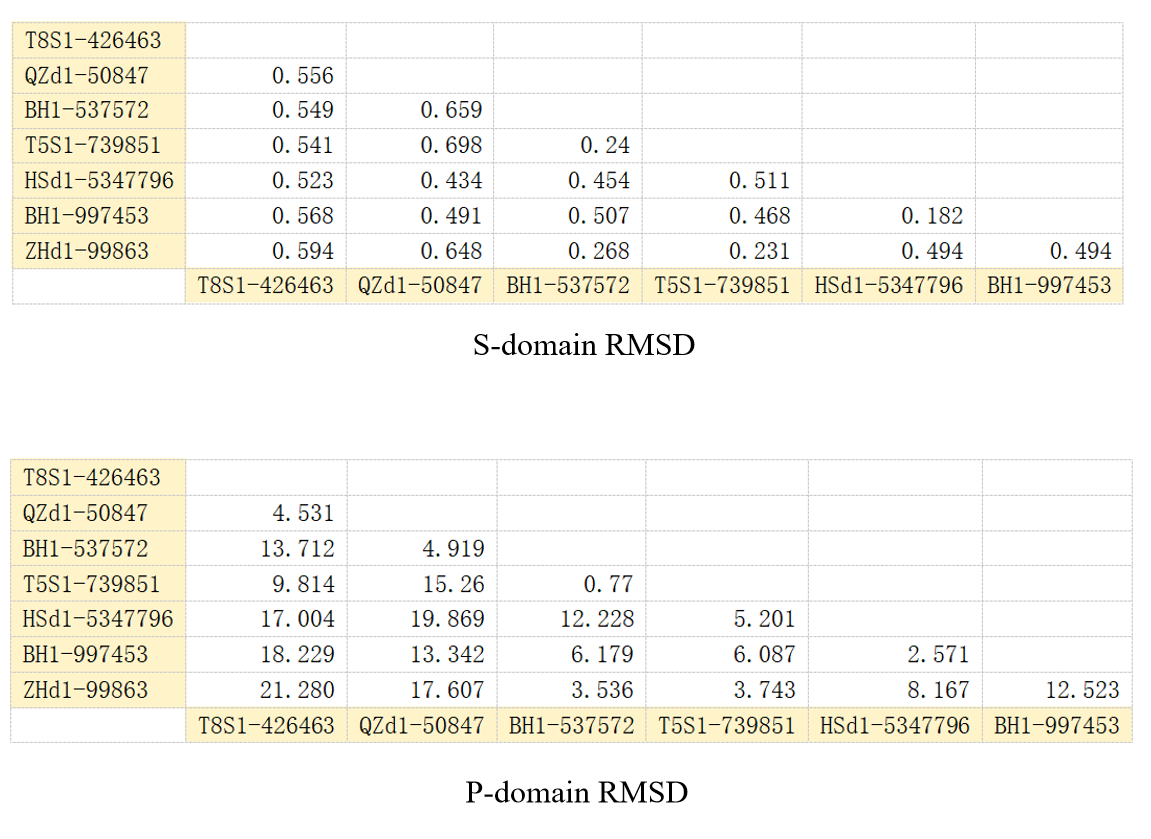


Figure S3 RMSD values of the S-domain and P-domain capsid proteins of Crucivirus related to oysters.


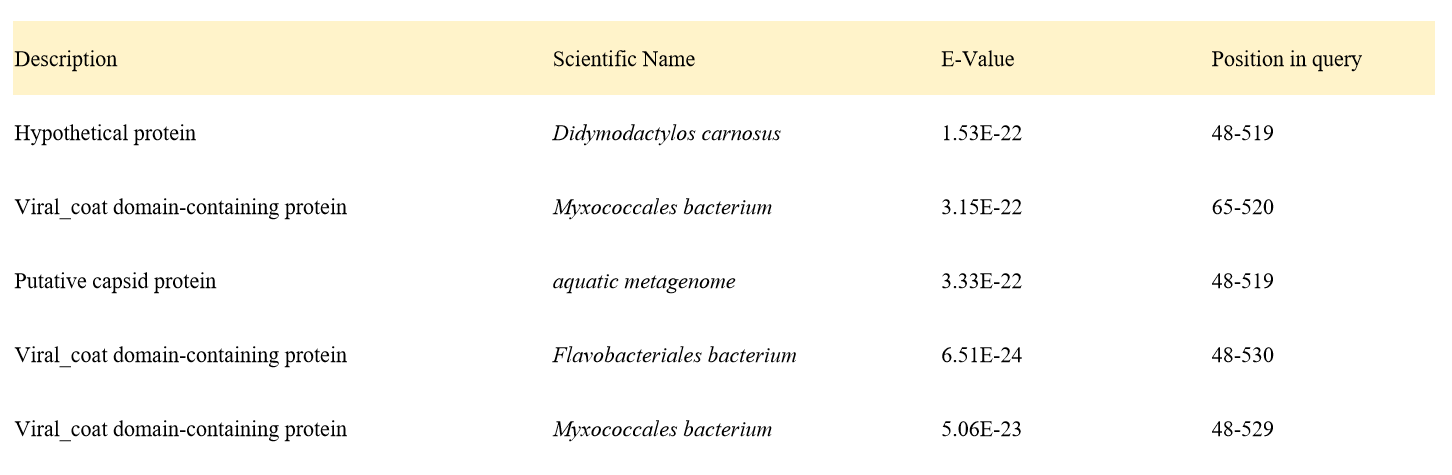


Figure S4 Top 5 species in terms of comparison and scoring of the three-dimensional structure of Crucivirus related to oysters.


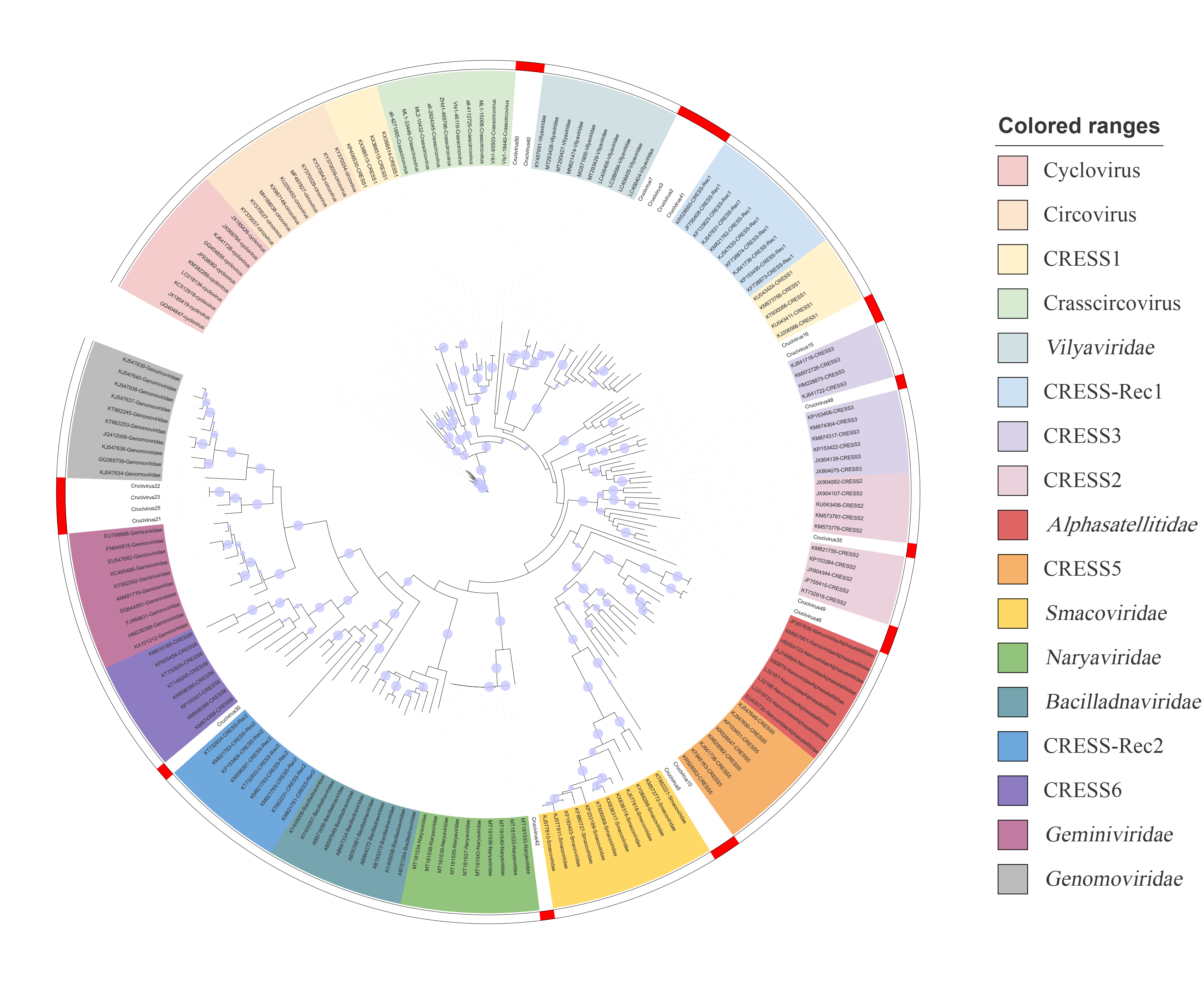


Figure S5 The phylogenetic tree of crucivirus and CRESS DNA viruses from different groups only shows branch lengths with a confidence level greater than 70, and the color of branch ends represents CRESS DNA viruses from different groups. The red square represents crucivirus.
